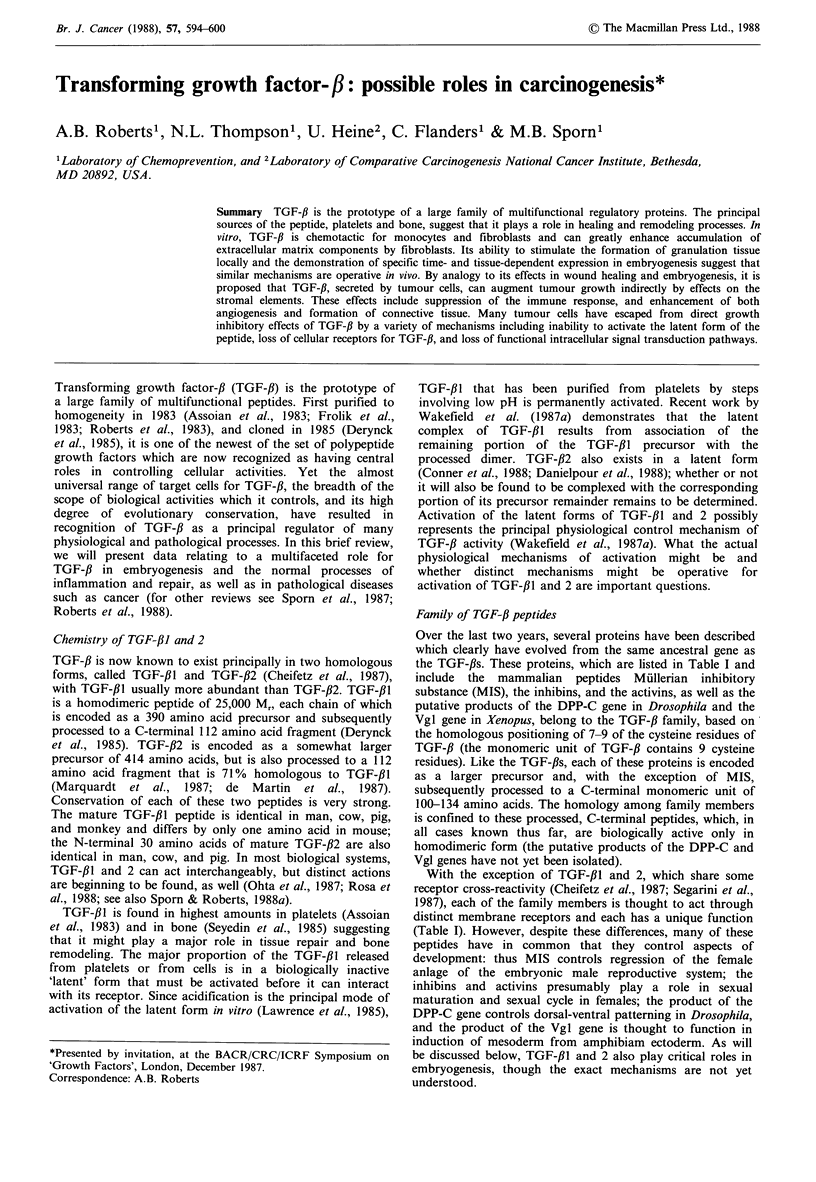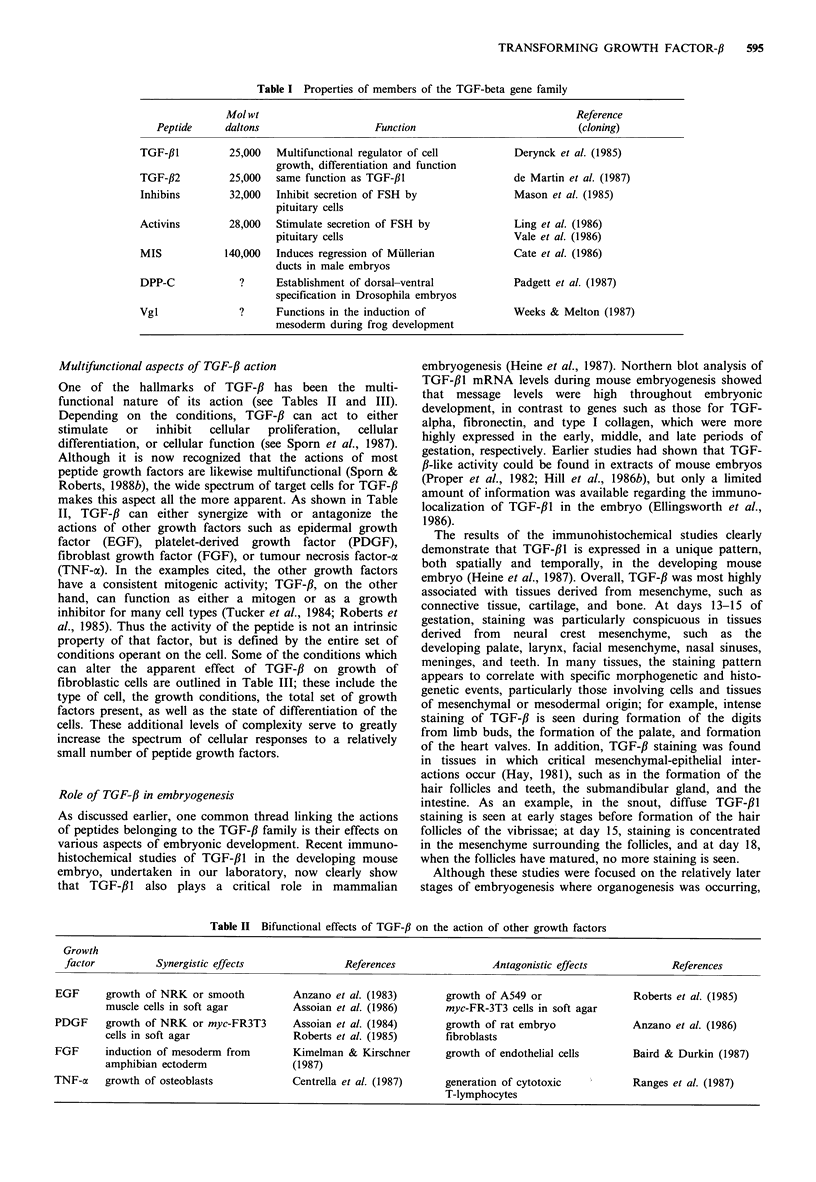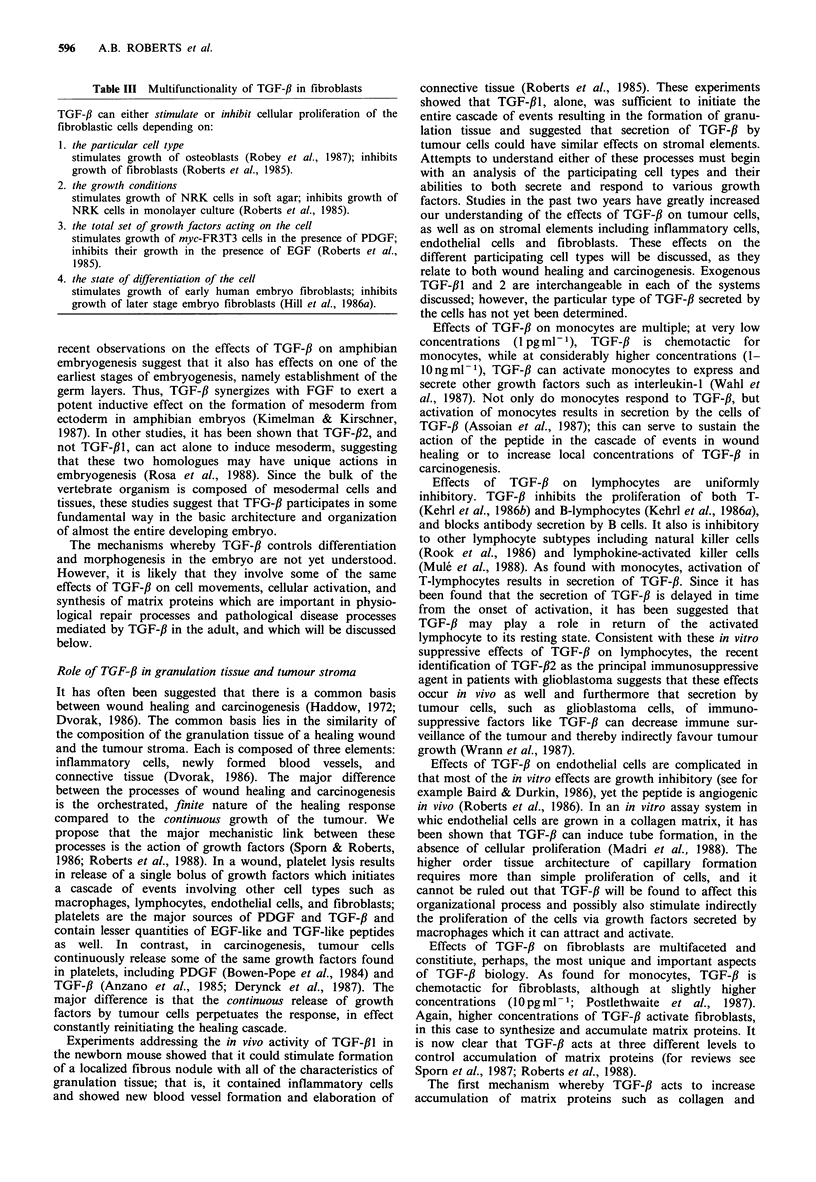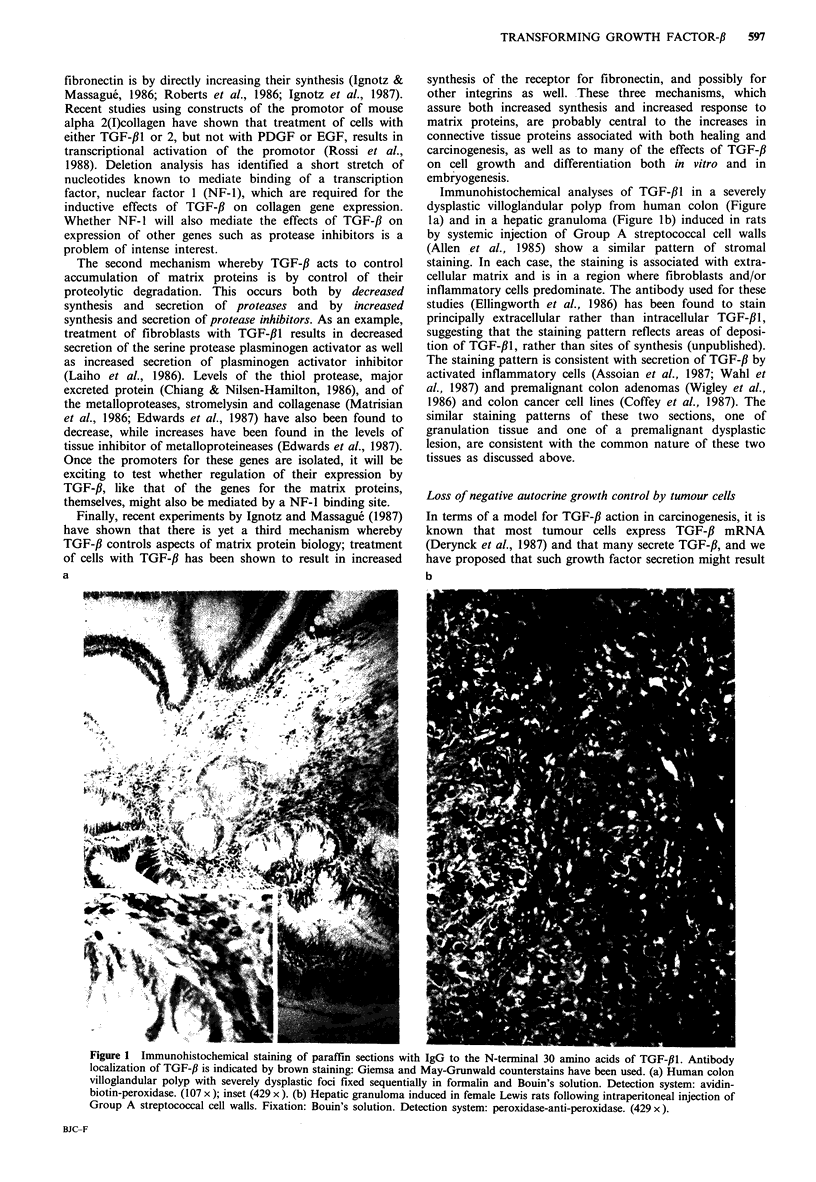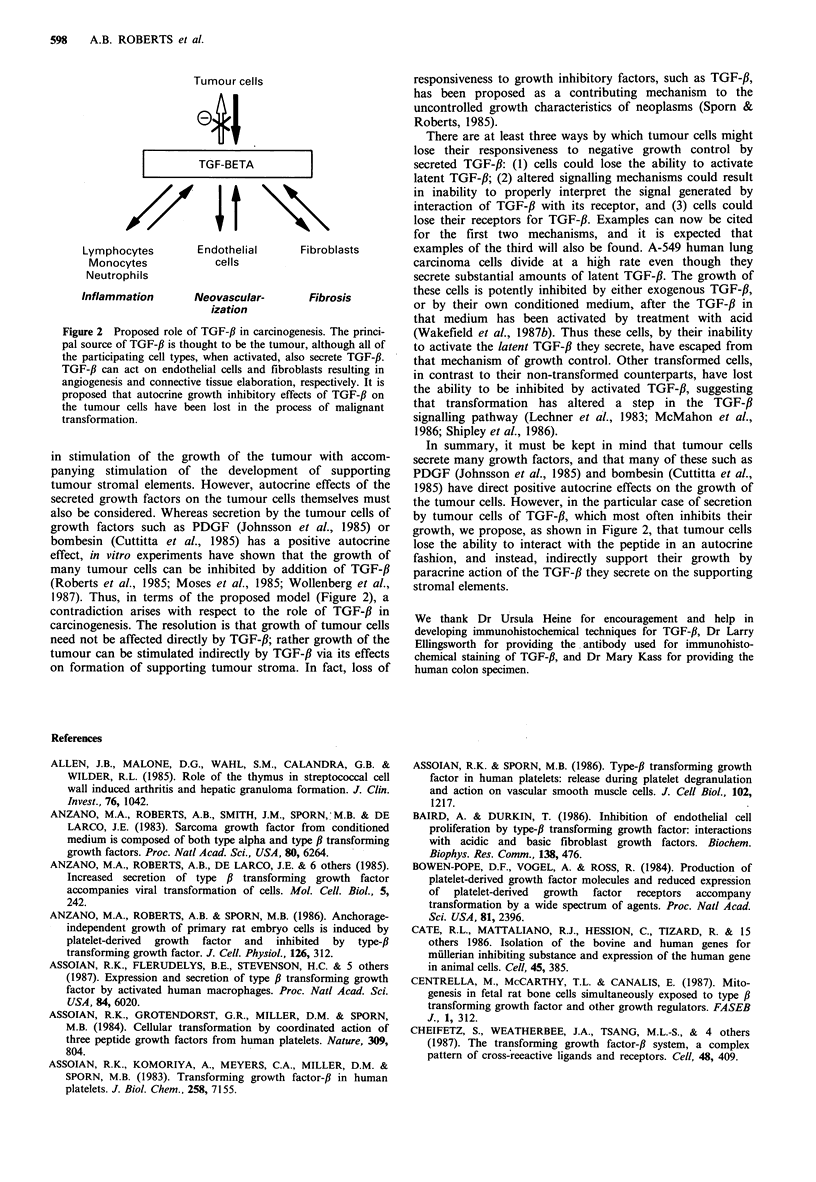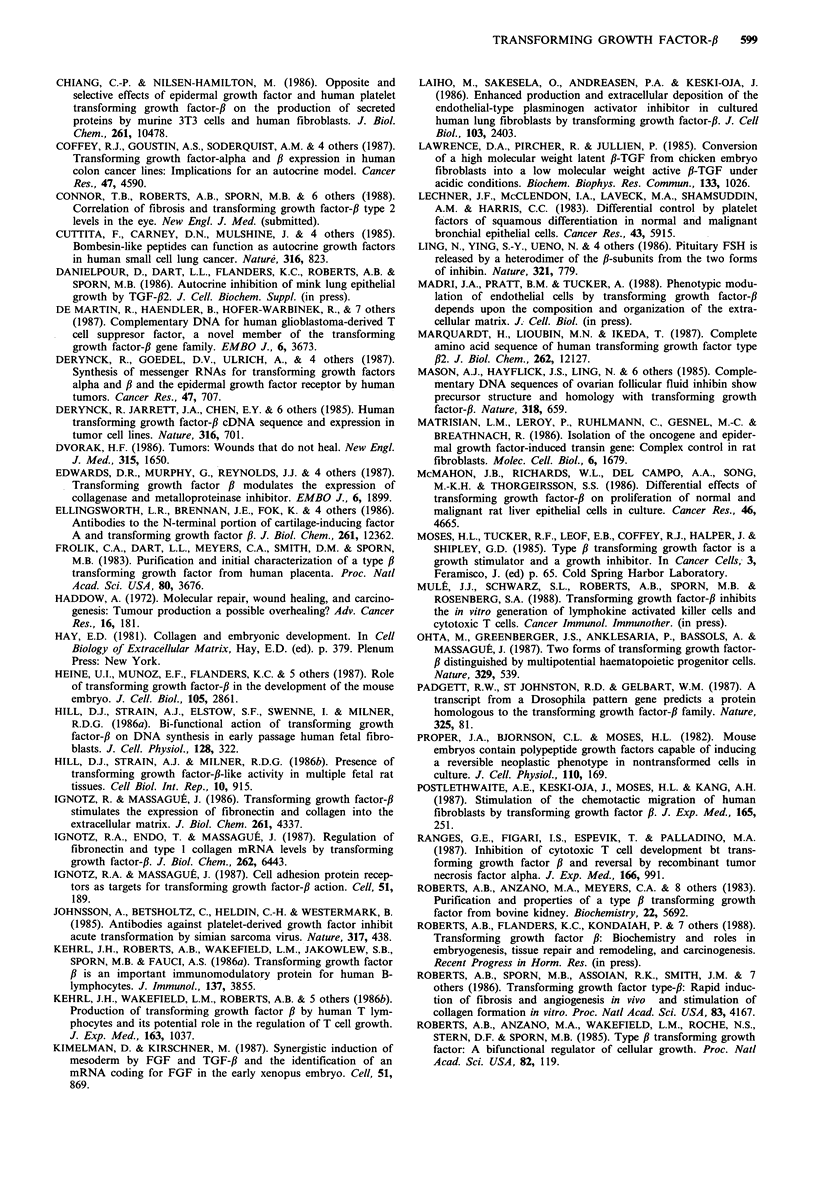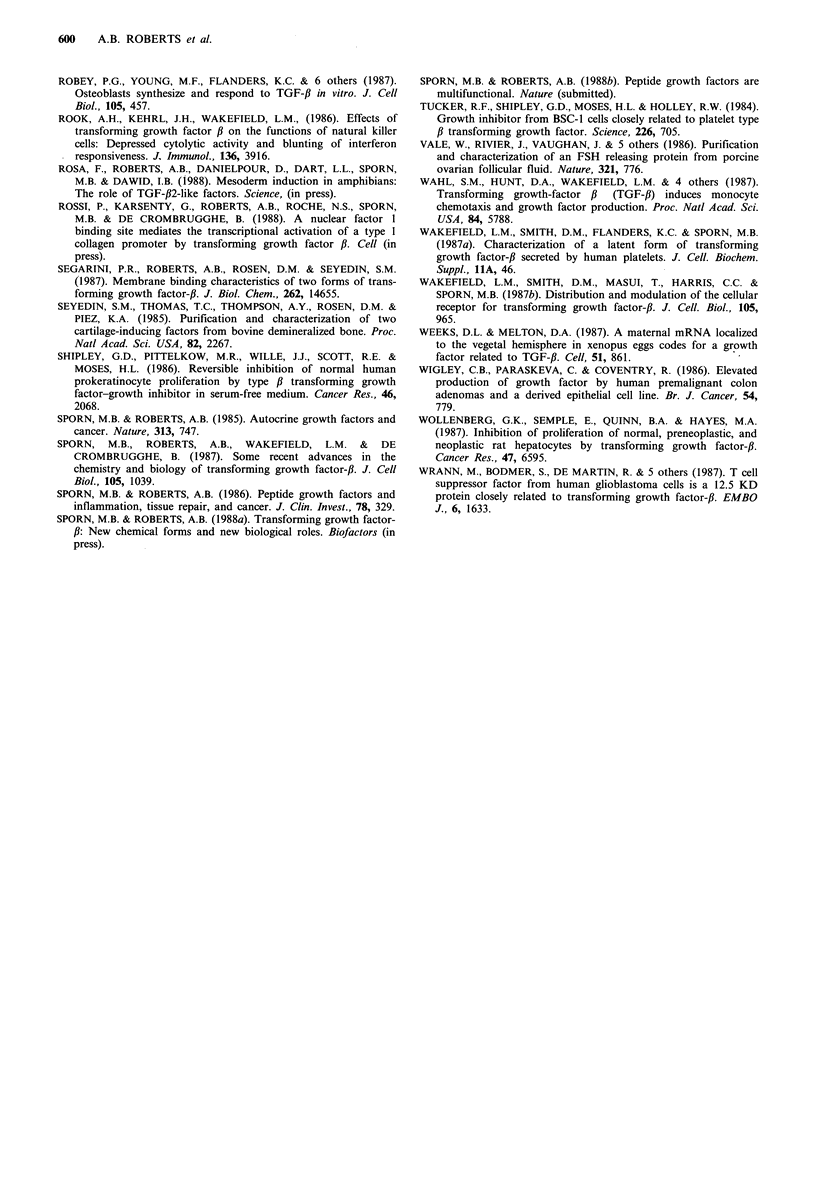# Transforming growth factor-beta: possible roles in carcinogenesis.

**DOI:** 10.1038/bjc.1988.135

**Published:** 1988-06

**Authors:** A. B. Roberts, N. L. Thompson, U. Heine, C. Flanders, M. B. Sporn

**Affiliations:** Laboratory of Chemoprevention, National Cancer Institute, Bethesda, MD 20892.

## Abstract

**Images:**


*Br. J. Cancer* (1988), **57**, 594–600
© The Macmillan Press Ltd., 1988

### Transforming growth factor-$\beta$: possible roles in carcinogenesis*

A.B. Roberts[1], N.L. Thompson[1], U. Heine[2], C. Flanders[1] & M.B. Sporn[1]

[1]*Laboratory of Chemoprevention, and* [2]*Laboratory of Comparative Carcinogenesis National Cancer Institute, Bethesda, MD 20892, USA.*

**Summary** TGF-$\beta$ is the prototype of a large family of multifunctional regulatory proteins. The principal sources of the peptide, platelets and bone, suggest that it plays a role in healing and remodeling processes. *In vitro*, TGF-$\beta$ is chemotactic for monocytes and fibroblasts and can greatly enhance accumulation of extracellular matrix components by fibroblasts. Its ability to stimulate the formation of granulation tissue locally and the demonstration of specific time- and tissue-dependent expression in embryogenesis suggest that similar mechanisms are operative *in vivo*. By analogy to its effects in wound healing and embryogenesis, it is proposed that TGF-$\beta$, secreted by tumour cells, can augment tumour growth indirectly by effects on the stromal elements. These effects include suppression of the immune response, and enhancement of both angiogenesis and formation of connective tissue. Many tumour cells have escaped from direct growth inhibitory effects of TGF-$\beta$ by a variety of mechanisms including inability to activate the latent form of the peptide, loss of cellular receptors for TGF-$\beta$, and loss of functional intracellular signal transduction pathways.

Transforming growth factor-$\beta$ (TGF-$\beta$) is the prototype of a large family of multifunctional peptides. First purified to homogeneity in 1983 (Assoian *et al.*, 1983; Frolik *et al.*, 1983; Roberts *et al.*, 1983), and cloned in 1985 (Derynck *et al.*, 1985), it is one of the newest of the set of polypeptide growth factors which are now recognized as having central roles in controlling cellular activities. Yet the almost universal range of target cells for TGF-$\beta$, the breadth of the scope of biological activities which it controls, and its high degree of evolutionary conservation, have resulted in recognition of TGF-$\beta$ as a principal regulator of many physiological and pathological processes. In this brief review, we will present data relating to a multifaceted role for TGF-$\beta$ in embryogenesis and the normal processes of inflammation and repair, as well as in pathological diseases such as cancer (for other reviews see Sporn *et al.*, 1987; Roberts *et al.*, 1988).

*Chemistry of TGF-$\beta$1 and 2*

TGF-$\beta$ is now known to exist principally in two homologous forms, called TGF-$\beta$1 and TGF-$\beta$2 (Cheifetz *et al.*, 1987), with TGF-$\beta$1 usually more abundant than TGF-$\beta$2. TGF-$\beta$1 is a homodimeric peptide of 25,000 $M_r$, each chain of which is encoded as a 390 amino acid precursor and subsequently processed to a C-terminal 112 amino acid fragment (Derynck *et al.*, 1985). TGF-$\beta$2 is encoded as a somewhat larger precursor of 414 amino acids, but is also processed to a 112 amino acid fragment that is 71% homologous to TGF-$\beta$1 (Marquardt *et al.*, 1987; de Martin *et al.*, 1987). Conservation of each of these two peptides is very strong. The mature TGF-$\beta$1 peptide is identical in man, cow, pig, and monkey and differs by only one amino acid in mouse; the N-terminal 30 amino acids of mature TGF-$\beta$2 are also identical in man, cow, and pig. In most biological systems, TGF-$\beta$1 and 2 can act interchangeably, but distinct actions are beginning to be found, as well (Ohta *et al.*, 1987; Rosa *et al.*, 1988; see also Sporn & Roberts, 1988*a*).

TGF-$\beta$1 is found in highest amounts in platelets (Assoian *et al.*, 1983) and in bone (Seyedin *et al.*, 1985) suggesting that it might play a major role in tissue repair and bone remodeling. The major proportion of the TGF-$\beta$1 released from platelets or from cells is in a biologically inactive 'latent' form that must be activated before it can interact with its receptor. Since acidification is the principal mode of activation of the latent form *in vitro* (Lawrence *et al.*, 1985), TGF-$\beta$1 that has been purified from platelets by steps involving low pH is permanently activated. Recent work by Wakefield *et al.* (1987*a*) demonstrates that the latent complex of TGF-$\beta$1 results from association of the remaining portion of the TGF-$\beta$1 precursor with the processed dimer. TGF-$\beta$2 also exists in a latent form (Conner *et al.*, 1988; Danielpour *et al.*, 1988); whether or not it will also be found to be complexed with the corresponding portion of its precursor remainder remains to be determined. Activation of the latent forms of TGF-$\beta$1 and 2 possibly represents the principal physiological control mechanism of TGF-$\beta$ activity (Wakefield *et al.*, 1987*a*). What the actual physiological mechanisms of activation might be and whether distinct mechanisms might be operative for activation of TGF-$\beta$1 and 2 are important questions.

*Family of TGF-$\beta$ peptides*

Over the last two years, several proteins have been described which clearly have evolved from the same ancestral gene as the TGF-$\beta$s. These proteins, which are listed in Table I and include the mammalian peptides Müllerian inhibitory substance (MIS), the inhibins, and the activins, as well as the putative products of the DPP-C gene in *Drosophila* and the Vgl gene in *Xenopus*, belong to the TGF-$\beta$ family, based on the homologous positioning of 7–9 of the cysteine residues of TGF-$\beta$ (the monomeric unit of TGF-$\beta$ contains 9 cysteine residues). Like the TGF-$\beta$s, each of these proteins is encoded as a larger precursor and, with the exception of MIS, subsequently processed to a C-terminal monomeric unit of 100–134 amino acids. The homology among family members is confined to these processed, C-terminal peptides, which, in all cases known thus far, are biologically active only in homodimeric form (the putative products of the DPP-C and Vgl genes have not yet been isolated).

With the exception of TGF-$\beta$1 and 2, which share some receptor cross-reactivity (Cheifetz *et al.*, 1987; Segarini *et al.*, 1987), each of the family members is thought to act through distinct membrane receptors and each has a unique function (Table I). However, despite these differences, many of these peptides have in common that they control aspects of development: thus MIS controls regression of the female anlage of the embryonic male reproductive system; the inhibins and activins presumably play a role in sexual maturation and sexual cycle in females; the product of the DPP-C gene controls dorsal-ventral patterning in *Drosophila*, and the product of the Vgl gene is thought to function in induction of mesoderm from amphibiam ectoderm. As will be discussed below, TGF-$\beta$1 and 2 also play critical roles in embryogenesis, though the exact mechanisms are not yet understood.

*Presented by invitation, at the BACR/CRC/ICRF Symposium on 'Growth Factors', London, December 1987.

Correspondence: A.B. Roberts

**Table I** Properties of members of the TGF-beta gene family

| *Peptide* | *Mol wt daltons* | *Function* | *Reference (cloning)* |
|---|---|---|---|
| TGF-β1 | 25,000 | Multifunctional regulator of cell growth, differentiation and function | Derynck *et al.* (1985) |
| TGF-β2 | 25,000 | same function as TGF-β1 | de Martin *et al.* (1987) |
| Inhibins | 32,000 | Inhibit secretion of FSH by pituitary cells | Mason *et al.* (1985) |
| Activins | 28,000 | Stimulate secretion of FSH by pituitary cells | Ling *et al.* (1986)<br>Vale *et al.* (1986) |
| MIS | 140,000 | Induces regression of Müllerian ducts in male embryos | Cate *et al.* (1986) |
| DPP-C | ? | Establishment of dorsal–ventral specification in Drosophila embryos | Padgett *et al.* (1987) |
| Vgl | ? | Functions in the induction of mesoderm during frog development | Weeks & Melton (1987) |

### *Multifunctional aspects of TGF-β action*

One of the hallmarks of TGF-β has been the multifunctional nature of its action (see Tables II and III). Depending on the conditions, TGF-β can act to either stimulate or inhibit cellular proliferation, cellular differentiation, or cellular function (see Sporn *et al.*, 1987). Although it is now recognized that the actions of most peptide growth factors are likewise multifunctional (Sporn & Roberts, 1988*b*), the wide spectrum of target cells for TGF-β makes this aspect all the more apparent. As shown in Table II, TGF-β can either synergize with or antagonize the actions of other growth factors such as epidermal growth factor (EGF), platelet-derived growth factor (PDGF), fibroblast growth factor (FGF), or tumour necrosis factor-α (TNF-α). In the examples cited, the other growth factors have a consistent mitogenic activity; TGF-β, on the other hand, can function as either a mitogen or as a growth inhibitor for many cell types (Tucker *et al.*, 1984; Roberts *et al.*, 1985). Thus the activity of the peptide is not an intrinsic property of that factor, but is defined by the entire set of conditions operant on the cell. Some of the conditions which can alter the apparent effect of TGF-β on growth of fibroblastic cells are outlined in Table III; these include the type of cell, the growth conditions, the total set of growth factors present, as well as the state of differentiation of the cells. These additional levels of complexity serve to greatly increase the spectrum of cellular responses to a relatively small number of peptide growth factors.

### *Role of TGF-β in embryogenesis*

As discussed earlier, one common thread linking the actions of peptides belonging to the TGF-β family is their effects on various aspects of embryonic development. Recent immunohistochemical studies of TGF-β1 in the developing mouse embryo, undertaken in our laboratory, now clearly show that TGF-β1 also plays a critical role in mammalian embryogenesis (Heine *et al.*, 1987). Northern blot analysis of TGF-β1 mRNA levels during mouse embryogenesis showed that message levels were high throughout embryonic development, in contrast to genes such as those for TGF-alpha, fibronectin, and type I collagen, which were more highly expressed in the early, middle, and late periods of gestation, respectively. Earlier studies had shown that TGF-β-like activity could be found in extracts of mouse embryos (Proper *et al.*, 1982; Hill *et al.*, 1986*b*), but only a limited amount of information was available regarding the immunolocalization of TGF-β1 in the embryo (Ellingsworth *et al.*, 1986).

The results of the immunohistochemical studies clearly demonstrate that TGF-β1 is expressed in a unique pattern, both spatially and temporally, in the developing mouse embryo (Heine *et al.*, 1987). Overall, TGF-β was most highly associated with tissues derived from mesenchyme, such as connective tissue, cartilage, and bone. At days 13–15 of gestation, staining was particularly conspicuous in tissues derived from neural crest mesenchyme, such as the developing palate, larynx, facial mesenchyme, nasal sinuses, meninges, and teeth. In many tissues, the staining pattern appears to correlate with specific morphogenetic and histogenetic events, particularly those involving cells and tissues of mesenchymal or mesodermal origin; for example, intense staining of TGF-β is seen during formation of the digits from limb buds, the formation of the palate, and formation of the heart valves. In addition, TGF-β staining was found in tissues in which critical mesenchymal-epithelial interactions occur (Hay, 1981), such as in the formation of the hair follicles and teeth, the submandibular gland, and the intestine. As an example, in the snout, diffuse TGF-β1 staining is seen at early stages before formation of the hair follicles of the vibrissae; at day 15, staining is concentrated in the mesenchyme surrounding the follicles, and at day 18, when the follicles have matured, no more staining is seen.

Although these studies were focused on the relatively later stages of embryogenesis where organogenesis was occurring,

**Table II** Bifunctional effects of TGF-β on the action of other growth factors

| *Growth factor* | *Synergistic effects* | *References* | *Antagonistic effects* | *References* |
|---|---|---|---|---|
| EGF | growth of NRK or smooth muscle cells in soft agar | Anzano *et al.* (1983)<br>Assoian *et al.* (1986) | growth of A549 or *myc*-FR-3T3 cells in soft agar | Roberts *et al.* (1985) |
| PDGF | growth of NRK or *myc*-FR3T3 cells in soft agar | Assoian *et al.* (1984)<br>Roberts *et al.* (1985) | growth of rat embryo fibroblasts | Anzano *et al.* (1986) |
| FGF | induction of mesoderm from amphibian ectoderm | Kimelman & Kirschner (1987) | growth of endothelial cells | Baird & Durkin (1987) |
| TNF-α | growth of osteoblasts | Centrella *et al.* (1987) | generation of cytotoxic T-lymphocytes | Ranges *et al.* (1987) |

**Table III** Multifunctionality of TGF-β in fibroblasts

TGF-β can either *stimulate* or *inhibit* cellular proliferation of the fibroblastic cells depending on:

1. *the particular cell type*
   stimulates growth of osteoblasts (Robey *et al.*, 1987); inhibits growth of fibroblasts (Roberts *et al.*, 1985).
2. *the growth conditions*
   stimulates growth of NRK cells in soft agar; inhibits growth of NRK cells in monolayer culture (Roberts *et al.*, 1985).
3. *the total set of growth factors acting on the cell*
   stimulates growth of *myc*-FR3T3 cells in the presence of PDGF; inhibits their growth in the presence of EGF (Roberts *et al.*, 1985).
4. *the state of differentiation of the cell*
   stimulates growth of early human embryo fibroblasts; inhibits growth of later stage embryo fibroblasts (Hill *et al.*, 1986*a*).

recent observations on the effects of TGF-β on amphibian embryogenesis suggest that it also has effects on one of the earliest stages of embryogenesis, namely establishment of the germ layers. Thus, TGF-β synergizes with FGF to exert a potent inductive effect on the formation of mesoderm from ectoderm in amphibian embryos (Kimelman & Kirschner, 1987). In other studies, it has been shown that TGF-β2, and not TGF-β1, can act alone to induce mesoderm, suggesting that these two homologues may have unique actions in embryogenesis (Rosa *et al.*, 1988). Since the bulk of the vertebrate organism is composed of mesodermal cells and tissues, these studies suggest that TFG-β participates in some fundamental way in the basic architecture and organization of almost the entire developing embryo.

The mechanisms whereby TGF-β controls differentiation and morphogenesis in the embryo are not yet understood. However, it is likely that they involve some of the same effects of TGF-β on cell movements, cellular activation, and synthesis of matrix proteins which are important in physiological repair processes and pathological disease processes mediated by TGF-β in the adult, and which will be discussed below.

*Role of TGF-β in granulation tissue and tumour stroma*

It has often been suggested that there is a common basis between wound healing and carcinogenesis (Haddow, 1972; Dvorak, 1986). The common basis lies in the similarity of the composition of the granulation tissue of a healing wound and the tumour stroma. Each is composed of three elements: inflammatory cells, newly formed blood vessels, and connective tissue (Dvorak, 1986). The major difference between the processes of wound healing and carcinogenesis is the orchestrated, *finite* nature of the healing response compared to the *continuous* growth of the tumour. We propose that the major mechanistic link between these processes is the action of growth factors (Sporn & Roberts, 1986; Roberts *et al.*, 1988). In a wound, platelet lysis results in release of a single bolus of growth factors which initiates a cascade of events involving other cell types such as macrophages, lymphocytes, endothelial cells, and fibroblasts; platelets are the major sources of PDGF and TGF-β and contain lesser quantities of EGF-like and TGF-like peptides as well. In contrast, in carcinogenesis, tumour cells continuously release some of the same growth factors found in platelets, including PDGF (Bowen-Pope *et al.*, 1984) and TGF-β (Anzano *et al.*, 1985; Derynck *et al.*, 1987). The major difference is that the *continuous* release of growth factors by tumour cells perpetuates the response, in effect constantly reinitiating the healing cascade.

Experiments addressing the *in vivo* activity of TGF-β1 in the newborn mouse showed that it could stimulate formation of a localized fibrous nodule with all of the characteristics of granulation tissue; that is, it contained inflammatory cells and showed new blood vessel formation and elaboration of connective tissue (Roberts *et al.*, 1985). These experiments showed that TGF-β1, alone, was sufficient to initiate the entire cascade of events resulting in the formation of granulation tissue and suggested that secretion of TGF-β by tumour cells could have similar effects on stromal elements. Attempts to understand either of these processes must begin with an analysis of the participating cell types and their abilities to both secrete and respond to various growth factors. Studies in the past two years have greatly increased our understanding of the effects of TGF-β on tumour cells, as well as on stromal elements including inflammatory cells, endothelial cells and fibroblasts. These effects on the different participating cell types will be discussed, as they relate to both wound healing and carcinogenesis. Exogenous TGF-β1 and 2 are interchangeable in each of the systems discussed; however, the particular type of TGF-β secreted by the cells has not yet been determined.

Effects of TGF-β on monocytes are multiple; at very low concentrations ($1\,pg\,ml^{-1}$), TGF-β is chemotactic for monocytes, while at considerably higher concentrations (1–$10\,ng\,ml^{-1}$), TGF-β can activate monocytes to express and secrete other growth factors such as interleukin-1 (Wahl *et al.*, 1987). Not only do monocytes respond to TGF-β, but activation of monocytes results in secretion by the cells of TGF-β (Assoian *et al.*, 1987); this can serve to sustain the action of the peptide in the cascade of events in wound healing or to increase local concentrations of TGF-β in carcinogenesis.

Effects of TGF-β on lymphocytes are uniformly inhibitory. TGF-β inhibits the proliferation of both T- (Kehrl *et al.*, 1986*b*) and B-lymphocytes (Kehrl *et al.*, 1986*a*), and blocks antibody secretion by B cells. It also is inhibitory to other lymphocyte subtypes including natural killer cells (Rook *et al.*, 1986) and lymphokine-activated killer cells (Mulé *et al.*, 1988). As found with monocytes, activation of T-lymphocytes results in secretion of TGF-β. Since it has been found that the secretion of TGF-β is delayed in time from the onset of activation, it has been suggested that TGF-β may play a role in return of the activated lymphocyte to its resting state. Consistent with these *in vitro* suppressive effects of TGF-β on lymphocytes, the recent identification of TGF-β2 as the principal immunosuppressive agent in patients with glioblastoma suggests that these effects occur *in vivo* as well and furthermore that secretion by tumour cells, such as glioblastoma cells, of immunosuppressive factors like TGF-β can decrease immune surveillance of the tumour and thereby indirectly favour tumour growth (Wrann *et al.*, 1987).

Effects of TGF-β on endothelial cells are complicated in that most of the *in vitro* effects are growth inhibitory (see for example Baird & Durkin, 1986), yet the peptide is angiogenic *in vivo* (Roberts *et al.*, 1986). In an *in vitro* assay system in whic endothelial cells are grown in a collagen matrix, it has been shown that TGF-β can induce tube formation, in the absence of cellular proliferation (Madri *et al.*, 1988). The higher order tissue architecture of capillary formation requires more than simple proliferation of cells, and it cannot be ruled out that TGF-β will be found to affect this organizational process and possibly also stimulate indirectly the proliferation of the cells via growth factors secreted by macrophages which it can attract and activate.

Effects of TGF-β on fibroblasts are multifaceted and constitiute, perhaps, the most unique and important aspects of TGF-β biology. As found for monocytes, TGF-β is chemotactic for fibroblasts, although at slightly higher concentrations ($10\,pg\,ml^{-1}$; Postlethwaite *et al.*, 1987). Again, higher concentrations of TGF-β activate fibroblasts, in this case to synthesize and accumulate matrix proteins. It is now clear that TGF-β acts at three different levels to control accumulation of matrix proteins (for reviews see Sporn *et al.*, 1987; Roberts *et al.*, 1988).

The first mechanism whereby TGF-β acts to increase accumulation of matrix proteins such as collagen and fibronectin is by directly increasing their synthesis (Ignotz & Massagué, 1986; Roberts *et al*., 1986; Ignotz *et al*., 1987). Recent studies using constructs of the promotor of mouse alpha 2(I)collagen have shown that treatment of cells with either TGF-β1 or 2, but not with PDGF or EGF, results in transcriptional activation of the promotor (Rossi *et al*., 1988). Deletion analysis has identified a short stretch of nucleotides known to mediate binding of a transcription factor, nuclear factor 1 (NF-1), which are required for the inductive effects of TGF-β on collagen gene expression. Whether NF-1 will also mediate the effects of TGF-β on expression of other genes such as protease inhibitors is a problem of intense interest.

The second mechanism whereby TGF-β acts to control accumulation of matrix proteins is by control of their proteolytic degradation. This occurs both by *decreased* synthesis and secretion of *proteases* and by *increased* synthesis and secretion of *protease inhibitors*. As an example, treatment of fibroblasts with TGF-β1 results in decreased secretion of the serine protease plasminogen activator as well as increased secretion of plasminogen activator inhibitor (Laiho *et al*., 1986). Levels of the thiol protease, major excreted protein (Chiang & Nilsen-Hamilton, 1986), and of the metalloproteases, stromelysin and collagenase (Matrisian *et al*., 1986; Edwards *et al*., 1987) have also been found to decrease, while increases have been found in the levels of tissue inhibitor of metalloproteineases (Edwards *et al*., 1987). Once the promoters for these genes are isolated, it will be exciting to test whether regulation of their expression by TGF-β, like that of the genes for the matrix proteins, themselves, might also be mediated by a NF-1 binding site.

Finally, recent experiments by Ignotz and Massagué (1987) have shown that there is yet a third mechanism whereby TGF-β controls aspects of matrix protein biology; treatment of cells with TGF-β has been shown to result in increased synthesis of the receptor for fibronectin, and possibly for other integrins as well. These three mechanisms, which assure both increased synthesis and increased response to matrix proteins, are probably central to the increases in connective tissue proteins associated with both healing and carcinogenesis, as well as to many of the effects of TGF-β on cell growth and differentiation both *in vitro* and in embryogenesis.

Immunohistochemical analyses of TGF-β1 in a severely dysplastic villoglandular polyp from human colon (Figure 1a) and in a hepatic granuloma (Figure 1b) induced in rats by systemic injection of Group A streptococcal cell walls (Allen *et al*., 1985) show a similar pattern of stromal staining. In each case, the staining is associated with extracellular matrix and is in a region where fibroblasts and/or inflammatory cells predominate. The antibody used for these studies (Ellingworth *et al*., 1986) has been found to stain principally extracellular rather than intracellular TGF-β1, suggesting that the staining pattern reflects areas of deposition of TGF-β1, rather than sites of synthesis (unpublished). The staining pattern is consistent with secretion of TGF-β by activated inflammatory cells (Assoian *et al*., 1987; Wahl *et al*., 1987) and premalignant colon adenomas (Wigley *et al*., 1986) and colon cancer cell lines (Coffey *et al*., 1987). The similar staining patterns of these two sections, one of granulation tissue and one of a premalignant dysplastic lesion, are consistent with the common nature of these two tissues as discussed above.

### *Loss of negative autocrine growth control by tumour cells*

In terms of a model for TGF-β action in carcinogenesis, it is known that most tumour cells express TGF-β mRNA (Derynck *et al*., 1987) and that many secrete TGF-β, and we have proposed that such growth factor secretion might result a b

**Figure 1** Immunohistochemical staining of paraffin sections with IgG to the N-terminal 30 amino acids of TGF-β1. Antibody localization of TGF-β is indicated by brown staining: Giemsa and May-Grunwald counterstains have been used. (a) Human colon villoglandular polyp with severely dysplastic foci fixed sequentially in formalin and Bouin's solution. Detection system: avidin-biotin-peroxidase. (107 ×); inset (429 ×). (b) Hepatic granuloma induced in female Lewis rats following intraperitoneal injection of Group A streptococcal cell walls. Fixation: Bouin's solution. Detection system: peroxidase-anti-peroxidase. (429 ×).

**Figure 2** Proposed role of TGF-$\beta$ in carcinogenesis. The principal source of TGF-$\beta$ is thought to be the tumour, although all of the participating cell types, when activated, also secrete TGF-$\beta$. TGF-$\beta$ can act on endothelial cells and fibroblasts resulting in angiogenesis and connective tissue elaboration, respectively. It is proposed that autocrine growth inhibitory effects of TGF-$\beta$ on the tumour cells have been lost in the process of malignant transformation.

in stimulation of the growth of the tumour with accompanying stimulation of the development of supporting tumour stromal elements. However, autocrine effects of the secreted growth factors on the tumour cells themselves must also be considered. Whereas secretion by the tumour cells of growth factors such as PDGF (Johnsson *et al.*, 1985) or bombesin (Cuttitta *et al.*, 1985) has a positive autocrine effect, *in vitro* experiments have shown that the growth of many tumour cells can be inhibited by addition of TGF-$\beta$ (Roberts *et al.*, 1985; Moses *et al.*, 1985; Wollenberg *et al.*, 1987). Thus, in terms of the proposed model (Figure 2), a contradiction arises with respect to the role of TGF-$\beta$ in carcinogenesis. The resolution is that growth of tumour cells need not be affected directly by TGF-$\beta$; rather growth of the tumour can be stimulated indirectly by TGF-$\beta$ via its effects on formation of supporting tumour stroma. In fact, loss of responsiveness to growth inhibitory factors, such as TGF-$\beta$, has been proposed as a contributing mechanism to the uncontrolled growth characteristics of neoplasms (Sporn & Roberts, 1985).

There are at least three ways by which tumour cells might lose their responsiveness to negative growth control by secreted TGF-$\beta$: (1) cells could lose the ability to activate latent TGF-$\beta$; (2) altered signalling mechanisms could result in inability to properly interpret the signal generated by interaction of TGF-$\beta$ with its receptor, and (3) cells could lose their receptors for TGF-$\beta$. Examples can now be cited for the first two mechanisms, and it is expected that examples of the third will also be found. A-549 human lung carcinoma cells divide at a high rate even though they secrete substantial amounts of latent TGF-$\beta$. The growth of these cells is potently inhibited by either exogenous TGF-$\beta$, or by their own conditioned medium, after the TGF-$\beta$ in that medium has been activated by treatment with acid (Wakefield *et al.*, 1987*b*). Thus these cells, by their inability to activate the *latent* TGF-$\beta$ they secrete, have escaped from that mechanism of growth control. Other transformed cells, in contrast to their non-transformed counterparts, have lost the ability to be inhibited by activated TGF-$\beta$, suggesting that transformation has altered a step in the TGF-$\beta$ signalling pathway (Lechner *et al.*, 1983; McMahon *et al.*, 1986; Shipley *et al.*, 1986).

In summary, it must be kept in mind that tumour cells secrete many growth factors, and that many of these such as PDGF (Johnsson *et al.*, 1985) and bombesin (Cuttitta *et al.*, 1985) have direct positive autocrine effects on the growth of the tumour cells. However, in the particular case of secretion by tumour cells of TGF-$\beta$, which most often inhibits their growth, we propose, as shown in Figure 2, that tumour cells lose the ability to interact with the peptide in an autocrine fashion, and instead, indirectly support their growth by paracrine action of the TGF-$\beta$ they secrete on the supporting stromal elements.

We thank Dr Ursula Heine for encouragement and help in developing immunohistochemical techniques for TGF-$\beta$, Dr Larry Ellingsworth for providing the antibody used for immunohistochemical staining of TGF-$\beta$, and Dr Mary Kass for providing the human colon specimen.

### References

ALLEN, J.B., MALONE, D.G., WAHL, S.M., CALANDRA, G.B. & WILDER, R.L. (1985). Role of the thymus in streptococcal cell wall induced arthritis and hepatic granuloma formation. *J. Clin. Invest.*, **76**, 1042.

ANZANO, M.A., ROBERTS, A.B., SMITH, J.M., SPORN, M.B. & DE LARCO, J.E. (1983). Sarcoma growth factor from conditioned medium is composed of both type alpha and type $\beta$ transforming growth factors. *Proc. Natl Acad. Sci., USA*, **80**, 6264.

ANZANO, M.A., ROBERTS, A.B., DE LARCO, J.E. & 6 others (1985). Increased secretion of type $\beta$ transforming growth factor accompanies viral transformation of cells. *Mol. Cell. Biol.*, **5**, 242.

ANZANO, M.A., ROBERTS, A.B. & SPORN, M.B. (1986). Anchorage-independent growth of primary rat embryo cells is induced by platelet-derived growth factor and inhibited by type-$\beta$ transforming growth factor. *J. Cell. Physiol.*, **126**, 312.

ASSOIAN, R.K., FLERUDELYS, B.E., STEVENSON, H.C. & 5 others (1987). Expression and secretion of type $\beta$ transforming growth factor by activated human macrophages. *Proc. Natl Acad. Sci. USA*, **84**, 6020.

ASSOIAN, R.K., GROTENDORST, G.R., MILLER, D.M. & SPORN, M.B. (1984). Cellular transformation by coordinated action of three peptide growth factors from human platelets. *Nature*, **309**, 804.

ASSOIAN, R.K., KOMORIYA, A., MEYERS, C.A., MILLER, D.M. & SPORN, M.B. (1983). Transforming growth factor-$\beta$ in human platelets. *J. Biol. Chem.*, **258**, 7155.

ASSOIAN, R.K. & SPORN, M.B. (1986). Type-$\beta$ transforming growth factor in human platelets: release during platelet degranulation and action on vascular smooth muscle cells. *J. Cell Biol.*, **102**, 1217.

BAIRD, A. & DURKIN, T. (1986). Inhibition of endothelial cell proliferation by type-$\beta$ transforming growth factor: interactions with acidic and basic fibroblast growth factors. *Biochem. Biophys. Res. Comm.*, **138**, 476.

BOWEN-POPE, D.F., VOGEL, A. & ROSS, R. (1984). Production of platelet-derived growth factor molecules and reduced expression of platelet-derived growth factor receptors accompany transformation by a wide spectrum of agents. *Proc. Natl Acad. Sci. USA*, **81**, 2396.

CATE, R.L., MATTALIANO, R.J., HESSION, C., TIZARD, R. & 15 others 1986. Isolation of the bovine and human genes for müllerian inhibiting substance and expression of the human gene in animal cells. *Cell*, **45**, 385.

CENTRELLA, M., McCARTHY, T.L. & CANALIS, E. (1987). Mitogenesis in fetal rat bone cells simultaneously exposed to type $\beta$ transforming growth factor and other growth regulators. *FASEB J.*, **1**, 312.

CHEIFETZ, S., WEATHERBEE, J.A., TSANG, M.L.-S., & 4 others (1987). The transforming growth factor-$\beta$ system, a complex pattern of cross-reeactive ligands and receptors. *Cell*, **48**, 409.

CHIANG, C.-P. & NILSEN-HAMILTON, M. (1986). Opposite and selective effects of epidermal growth factor and human platelet transforming growth factor-β on the production of secreted proteins by murine 3T3 cells and human fibroblasts. *J. Biol. Chem.*, **261**, 10478.

COFFEY, R.J., GOUSTIN, A.S., SODERQUIST, A.M. & 4 others (1987). Transforming growth factor-alpha and β expression in human colon cancer lines: Implications for an autocrine model. *Cancer Res.*, **47**, 4590.

CONNOR, T.B., ROBERTS, A.B., SPORN, M.B. & 6 others (1988). Correlation of fibrosis and transforming growth factor-β type 2 levels in the eye. *New Engl. J. Med.* (submitted).

CUTTITA, F., CARNEY, D.N., MULSHINE, J. & 4 others (1985). Bombesin-like peptides can function as autocrine growth factors in human small cell lung cancer. *Nature*, **316**, 823.

DANIELPOUR, D., DART, L.L., FLANDERS, K.C., ROBERTS, A.B. & SPORN, M.B. (1986). Autocrine inhibition of mink lung epithelial growth by TGF-β2. *J. Cell. Biochem. Suppl.* (in press).

DE MARTIN, R., HAENDLER, B., HOFER-WARBINEK, R., & 7 others (1987). Complementary DNA for human glioblastoma-derived T cell suppresor factor, a novel member of the transforming growth factor-β gene family. *EMBO J.*, **6**, 3673.

DERYNCK, R., GOEDEL, D.V., ULRICH, A., & 4 others (1987). Synthesis of messenger RNAs for transforming growth factors alpha and β and the epidermal growth factor receptor by human tumors. *Cancer Res.*, **47**, 707.

DERYNCK, R. JARRETT, J.A., CHEN, E.Y. & 6 others (1985). Human transforming growth factor-β cDNA sequence and expression in tumor cell lines. *Nature*, **316**, 701.

DVORAK, H.F. (1986). Tumors: Wounds that do not heal. *New Engl. J. Med.*, **315**, 1650.

EDWARDS, D.R., MURPHY, G., REYNOLDS, J.J. & 4 others (1987). Transforming growth factor β modulates the expression of collagenase and metalloproteinase inhibitor. *EMBO J.*, **6**, 1899.

ELLINGSWORTH, L.R., BRENNAN, J.E., FOK, K. & 4 others (1986). Antibodies to the N-terminal portion of cartilage-inducing factor A and transforming growth factor β. *J. Biol. Chem.*, **261**, 12362.

FROLIK, C.A., DART, L.L., MEYERS, C.A., SMITH, D.M. & SPORN, M.B. (1983). Purification and initial characterization of a type β transforming growth factor from human placenta. *Proc. Natl Acad. Sci. USA*, **80**, 3676.

HADDOW, A. (1972). Molecular repair, wound healing, and carcinogenesis: Tumour production a possible overhealing? *Adv. Cancer Res.*, **16**, 181.

HAY, E.D. (1981). Collagen and embryonic development. In *Cell Biology of Extracellular Matrix*, Hay, E.D. (ed). p. 379. Plenum Press: New York.

HEINE, U.I., MUNOZ, E.F., FLANDERS, K.C. & 5 others (1987). Role of transforming growth factor-β in the development of the mouse embryo. *J. Cell. Biol.*, **105**, 2861.

HILL, D.J., STRAIN, A.J., ELSTOW, S.F., SWENNE, I. & MILNER, R.D.G. (1986*a*). Bi-functional action of transforming growth factor-β on DNA synthesis in early passage human fetal fibroblasts. *J. Cell. Physiol.*, **128**, 322.

HILL, D.J., STRAIN, A.J. & MILNER, R.D.G. (1986*b*). Presence of transforming growth factor-β-like activity in multiple fetal rat tissues. *Cell Biol. Int. Rep.*, **10**, 915.

IGNOTZ, R. & MASSAGUÉ, J. (1986). Transforming growth factor-β stimulates the expression of fibronectin and collagen into the extracellular matrix. *J. Biol. Chem.* **261**, 4337.

IGNOTZ, R.A., ENDO, T. & MASSAGUÉ, J. (1987). Regulation of fibronectin and type 1 collagen mRNA levels by transforming growth factor-β. *J. Biol. Chem.*, **262**, 6443.

IGNOTZ, R.A. & MASSAGUÉ, J. (1987). Cell adhesion protein receptors as targets for transforming growth factor-β action. *Cell*, **51**, 189.

JOHNSSON, A., BETSHOLTZ, C., HELDIN, C.-H. & WESTERMARK, B. (1985). Antibodies against platelet-derived growth factor inhibit acute transformation by simian sarcoma virus. *Nature*, **317**, 438.

KEHRL, J.H., ROBERTS, A.B., WAKEFIELD, L.M., JAKOWLEW, S.B., SPORN, M.B. & FAUCI, A.S. (1986*a*). Transforming growth factor β is an important immunomodulatory protein for human B-lymphocytes. *J. Immunol.*, **137**, 3855.

KEHRL, J.H., WAKEFIELD, L.M., ROBERTS, A.B. & 5 others (1986*b*). Production of transforming growth factor β by human T lymphocytes and its potential role in the regulation of T cell growth. *J. Exp. Med.*, **163**, 1037.

KIMELMAN, D. & KIRSCHNER, M. (1987). Synergistic induction of mesoderm by FGF and TGF-β and the identification of an mRNA coding for FGF in the early xenopus embryo. *Cell*, **51**, 869.

LAIHO, M., SAKESELA, O., ANDREASEN, P.A. & KESKI-OJA, J. (1986). Enhanced production and extracellular deposition of the endothelial-type plasminogen activator inhibitor in cultured human lung fibroblasts by transforming growth factor-β. *J. Cell Biol.*, **103**, 2403.

LAWRENCE, D.A., PIRCHER, R. & JULLIEN, P. (1985). Conversion of a high molecular weight latent β-TGF from chicken embryo fibroblasts into a low molecular weight active β-TGF under acidic conditions. *Biochem. Biophys. Res. Commun.*, **133**, 1026.

LECHNER, J.F., McCLENDON, I.A., LAVECK, M.A., SHAMSUDDIN, A.M. & HARRIS, C.C. (1983). Differential control by platelet factors of squamous differentiation in normal and malignant bronchial epithelial cells. *Cancer Res.*, **43**, 5915.

LING, N., YING, S.-Y., UENO, N. & 4 others (1986). Pituitary FSH is released by a heterodimer of the β-subunits from the two forms of inhibin. *Nature*, **321**, 779.

MADRI, J.A., PRATT, B.M. & TUCKER, A. (1988). Phenotypic modulation of endothelial cells by transforming growth factor-β depends upon the composition and organization of the extracellular matrix. *J. Cell. Biol.* (in press).

MARQUARDT, H., LIOUBIN, M.N. & IKEDA, T. (1987). Complete amino acid sequence of human transforming growth factor type β2. *J. Biol. Chem.*, **262**, 12127.

MASON, A.J., HAYFLICK, J.S., LING, N. & 6 others (1985). Complementary DNA sequences of ovarian follicular fluid inhibin show precursor structure and homology with transforming growth factor-β. *Nature*, **318**, 659.

MATRISIAN, L.M., LEROY, P., RUHLMANN, C., GESNEL, M.-C. & BREATHNACH, R. (1986). Isolation of the oncogene and epidermal growth factor-induced transin gene: Complex control in rat fibroblasts. *Molec. Cell. Biol.*, **6**, 1679.

McMAHON, J.B., RICHARDS, W.L., DEL CAMPO, A.A., SONG, M.-K.H. & THORGEIRSSON, S.S. (1986). Differential effects of transforming growth factor-β on proliferation of normal and malignant rat liver epithelial cells in culture. *Cancer Res.*, **46**, 4665.

MOSES, H.L., TUCKER, R.F., LEOF, E.B., COFFEY, R.J., HALPER, J. & SHIPLEY, G.D. (1985). Type β transforming growth factor is a growth stimulator and a growth inhibitor. In *Cancer Cells*, **3**, Feramisco, J. (ed) p. 65. Cold Spring Harbor Laboratory.

MULÉ, J.J., SCHWARZ, S.L., ROBERTS, A.B., SPORN, M.B. & ROSENBERG, S.A. (1988). Transforming growth factor-β inhibits the *in vitro* generation of lymphokine activated killer cells and cytotoxic T cells. *Cancer Immunol. Immunother.* (in press).

OHTA, M., GREENBERGER, J.S., ANKLESARIA, P., BASSOLS, A. & MASSAGUÉ, J. (1987). Two forms of transforming growth factor-β distinguished by multipotential haematopoietic progenitor cells. *Nature*, **329**, 539.

PADGETT, R.W., ST JOHNSTON, R.D. & GELBART, W.M. (1987). A transcript from a Drosophila pattern gene predicts a protein homologous to the transforming growth factor-β family. *Nature*, **325**, 81.

PROPER, J.A., BJORNSON, C.L. & MOSES, H.L. (1982). Mouse embryos contain polypeptide growth factors capable of inducing a reversible neoplastic phenotype in nontransformed cells in culture. *J. Cell. Physiol.*, **110**, 169.

POSTLETHWAITE, A.E., KESKI-OJA, J., MOSES, H.L. & KANG, A.H. (1987). Stimulation of the chemotactic migration of human fibroblasts by transforming growth factor β. *J. Exp. Med.*, **165**, 251.

RANGES, G.E., FIGARI, I.S., ESPEVIK, T. & PALLADINO, M.A. (1987). Inhibition of cytotoxic T cell development bt transforming growth factor β and reversal by recombinant tumor necrosis factor alpha. *J. Exp. Med.*, **166**, 991.

ROBERTS, A.B., ANZANO, M.A., MEYERS, C.A. & 8 others (1983). Purification and properties of a type β transforming growth factor from bovine kidney. *Biochemistry*, **22**, 5692.

ROBERTS, A.B., FLANDERS, K.C., KONDAIAH, P. & 7 others (1988). Transforming growth factor β: Biochemistry and roles in embryogenesis, tissue repair and remodeling, and carcinogenesis. *Recent Progress in Horm. Res.* (in press).

ROBERTS, A.B., SPORN, M.B., ASSOIAN, R.K., SMITH, J.M. & 7 others (1986). Transforming growth factor type-β: Rapid induction of fibrosis and angiogenesis *in vivo* and stimulation of collagen formation *in vitro*. *Proc. Natl Acad. Sci. USA*, **83**, 4167.

ROBERTS, A.B., ANZANO, M.A., WAKEFIELD, L.M., ROCHE, N.S., STERN, D.F. & SPORN, M.B. (1985). Type β transforming growth factor: A bifunctional regulator of cellular growth. *Proc. Natl Acad. Sci. USA*, **82**, 119.

ROBEY, P.G., YOUNG, M.F., FLANDERS, K.C. & 6 others (1987). Osteoblasts synthesize and respond to TGF-$\beta$ *in vitro*. *J. Cell Biol.*, **105**, 457.

ROOK, A.H., KEHRL, J.H., WAKEFIELD, L.M., (1986). Effects of transforming growth factor $\beta$ on the functions of natural killer cells: Depressed cytolytic activity and blunting of interferon responsiveness. *J. Immunol.*, **136**, 3916.

ROSA, F., ROBERTS, A.B., DANIELPOUR, D., DART, L.L., SPORN, M.B. & DAWID, I.B. (1988). Mesoderm induction in amphibians: The role of TGF-$\beta$2-like factors. *Science*, (in press).

ROSSI, P., KARSENTY, G., ROBERTS, A.B., ROCHE, N.S., SPORN, M.B. & DE CROMBRUGGHE, B. (1988). A nuclear factor 1 binding site mediates the transcriptional activation of a type 1 collagen promoter by transforming growth factor $\beta$. *Cell* (in press).

SEGARINI, P.R., ROBERTS, A.B., ROSEN, D.M. & SEYEDIN, S.M. (1987). Membrane binding characteristics of two forms of transforming growth factor-$\beta$. *J. Biol. Chem.*, **262**, 14655.

SEYEDIN, S.M., THOMAS, T.C., THOMPSON, A.Y., ROSEN, D.M. & PIEZ, K.A. (1985). Purification and characterization of two cartilage-inducing factors from bovine demineralized bone. *Proc. Natl Acad. Sci. USA*, **82**, 2267.

SHIPLEY, G.D., PITTELKOW, M.R., WILLE, J.J., SCOTT, R.E. & MOSES, H.L. (1986). Reversible inhibition of normal human prokeratinocyte proliferation by type $\beta$ transforming growth factor–growth inhibitor in serum-free medium. *Cancer Res.*, **46**, 2068.

SPORN, M.B. & ROBERTS, A.B. (1985). Autocrine growth factors and cancer. *Nature*, **313**, 747.

SPORN, M.B., ROBERTS, A.B., WAKEFIELD, L.M. & DE CROMBRUGGHE, B. (1987). Some recent advances in the chemistry and biology of transforming growth factor-$\beta$. *J. Cell Biol.*, **105**, 1039.

SPORN, M.B. & ROBERTS, A.B. (1986). Peptide growth factors and inflammation, tissue repair, and cancer. *J. Clin. Invest.*, **78**, 329.

SPORN, M.B. & ROBERTS, A.B. (1988*a*). Transforming growth factor-$\beta$: New chemical forms and new biological roles. *Biofactors* (in press).

SPORN, M.B. & ROBERTS, A.B. (1988*b*). Peptide growth factors are multifunctional. *Nature* (submitted).

TUCKER, R.F., SHIPLEY, G.D., MOSES, H.L. & HOLLEY, R.W. (1984). Growth inhibitor from BSC-1 cells closely related to platelet type $\beta$ transforming growth factor. *Science*, **226**, 705.

VALE, W., RIVIER, J., VAUGHAN, J. & 5 others (1986). Purification and characterization of an FSH releasing protein from porcine ovarian follicular fluid. *Nature*, **321**, 776.

WAHL, S.M., HUNT, D.A., WAKEFIELD, L.M. & 4 others (1987). Transforming growth-factor $\beta$ (TGF-$\beta$) induces monocyte chemotaxis and growth factor production. *Proc. Natl Acad. Sci. USA*, **84**, 5788.

WAKEFIELD, L.M., SMITH, D.M., FLANDERS, K.C. & SPORN, M.B. (1987*a*). Characterization of a latent form of transforming growth factor-$\beta$ secreted by human platelets. *J. Cell. Biochem. Suppl.*, **11A**, 46.

WAKEFIELD, L.M., SMITH, D.M., MASUI, T., HARRIS, C.C. & SPORN, M.B. (1987*b*). Distribution and modulation of the cellular receptor for transforming growth factor-$\beta$. *J. Cell. Biol.*, **105**, 965.

WEEKS, D.L. & MELTON, D.A. (1987). A maternal mRNA localized to the vegetal hemisphere in xenopus eggs codes for a growth factor related to TGF-$\beta$. *Cell*, **51**, 861.

WIGLEY, C.B., PARASKEVA, C. & COVENTRY, R. (1986). Elevated production of growth factor by human premalignant colon adenomas and a derived epithelial cell line. *Br. J. Cancer*, **54**, 779.

WOLLENBERG, G.K., SEMPLE, E., QUINN, B.A. & HAYES, M.A. (1987). Inhibition of proliferation of normal, preneoplastic, and neoplastic rat hepatocytes by transforming growth factor-$\beta$. *Cancer Res.*, **47**, 6595.

WRANN, M., BODMER, S., DE MARTIN, R. & 5 others (1987). T cell suppressor factor from human glioblastoma cells is a 12.5 KD protein closely related to transforming growth factor-$\beta$. *EMBO J.*, **6**, 1633.